# A Review on Obesity Management through Natural Compounds and a Green Nanomedicine-Based Approach

**DOI:** 10.3390/molecules26113278

**Published:** 2021-05-28

**Authors:** Monika Bhardwaj, Poonam Yadav, Divya Vashishth, Kavita Sharma, Ajay Kumar, Jyoti Chahal, Sunita Dalal, Sudhir Kumar Kataria

**Affiliations:** 1 Department of Zoology, Maharshi Dayanand University, Rohtak 124001, India; bhardwajmoni92@gmail.com (M.B.); poonambalewa93@gmail.com (P.Y.); vashdiv20@gmail.com (D.V.); 2 Department of Zoology, Gaur Brahman Degree College, Rohtak 124001, India; kavitasharma11061986@gmail.com; 3 Department of Zoology, Maharaja Neempal Singh Government College, Bhiwani 127021, India; ajay14286@gmail.com; 4 Department of Zoology, Hindu Girls College, Sonipat 131001, India; chahaljyoti@yahoo.co.in; 5 Department of Biotechnology, Kurukshetra University, Kurukshetra 136119, India; sdalal@kuk.ac.in

**Keywords:** obesity, nanoencapsulation, secondary metabolites and adipogenesis

## Abstract

Obesity is a serious health complication in almost every corner of the world. Excessive weight gain results in the onset of several other health issues such as type II diabetes, cancer, respiratory diseases, musculoskeletal disorders (especially osteoarthritis), and cardiovascular diseases. As allopathic medications and derived pharmaceuticals are partially successful in overcoming this health complication, there is an incessant need to develop new alternative anti-obesity strategies with long term efficacy and less side effects. Plants harbor secondary metabolites such as phenolics, flavonoids, terpenoids and other specific compounds that have been shown to have effective anti-obesity properties. Nanoencapsulation of these secondary metabolites enhances the anti-obesity efficacy of these natural compounds due to their speculated property of target specificity and enhanced efficiency. These nanoencapsulated and naive secondary metabolites show anti-obesity properties mainly by inhibiting the lipid and carbohydrate metabolizing enzymes, suppression of adipogenesis and appetite, and enhancing energy metabolism. This review focuses on the plants and their secondary metabolites, along with their nanoencapsulation, that have anti-obesity effects, with their possible acting mechanisms, for better human health.

## 1. Introduction

**Figure 1:**
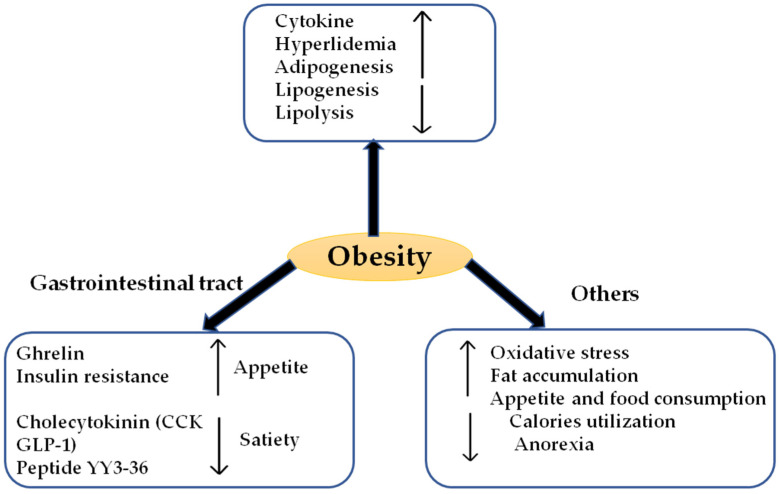
Obesity regulatory factors.

In past few decades, overweight and obese cases among people of all age groups have become a serious health issue. According to the WHO, obesity is mainly considered as excessive and abnormal fat accumulation that can lead to improper health functioning. Approximately 1.9 billion adults were overweight with a basal metabolic index (BMI ≥ 25) and among these, 650 million adults were obese (BMI ≥ 30), in 2016. The frequency of obesity almost tripled from 1975 to 2016. In 2019, an estimated 38.2 million children under 5 years old were obese all over the world and almost 50% of total children in Asia were obese or overweight [1]. Obesity is mainly due to genetic, environment and behavioral factors [2]. It mainly occurs due to an increase in the ratio of calories or energy intake to the calories or energy expenditure, which results from genetic susceptibility and lethargic lifestyle modifications [3]. Obesity and excessive weight gain can be controlled by modifications in diet and increased physical exercise, but these approaches are not rapid, so many people prefer chemical medications over these approaches for effective results [4]. Various chemical medications are available in the market that have anti-obesity property, such as orlistat, fenfluramine, coreaserin, rimonabant, cetlistat, sibutramine, and phentermine with topiramate, with different efficacy to control obesity [5,6]. These medications have several adverse effects on health like cardiometabolic abnormalities, anxiety, high blood pressure and pulse rate, and depressive disorders [7]. Therefore, more sophisticated efforts should be done to discover natural anti-obesity agents with less side effects and more efficiency. Accordingly, several natural secondary metabolites present in different plants like polyphenols, flavonoids, terpenoids, alkaloids, saponins, carboxylic acids, glycosides and tannins are reported to have anti-obesity efficacy through different mechanisms of action. Several bioactive compounds in edible plants, such as green tea with epigallocatechins, nobiletin in citrus peel, resveratrol, pterostilbene in berries, curcumin in turmeric, and anthocyanins in *Hibiscus sabdariffa*, are reported to suppress the obesity-causing factors [8,9]. Nanotechnology and nanoencapsulation of these secondary metabolites opens up a new horizon for overcoming this aforementioned health issue. These biochemicals can be encapsulated with biocompatible nanoparticles, which enhances their target specificity, bioavailability, stability and aqueous solubility [10]. The basic pathogenesis of obesity mainly includes an increase in appetite and a decrease in calorie expenditure by modulating physical activity and cellular functioning. These abnormalities increase the process of adipogenesis, which in turn increases the release of cytokines and vascular complications leading to cardiovascular system disorders like atherosclerosis and hyperlipidemia. The various factors responsible for the induction of obesity and other related health disorders are shown in Figure 1.

## 2. Role of Natural Products in Obesity: Regulation of Metabolism

**Figure 2:**
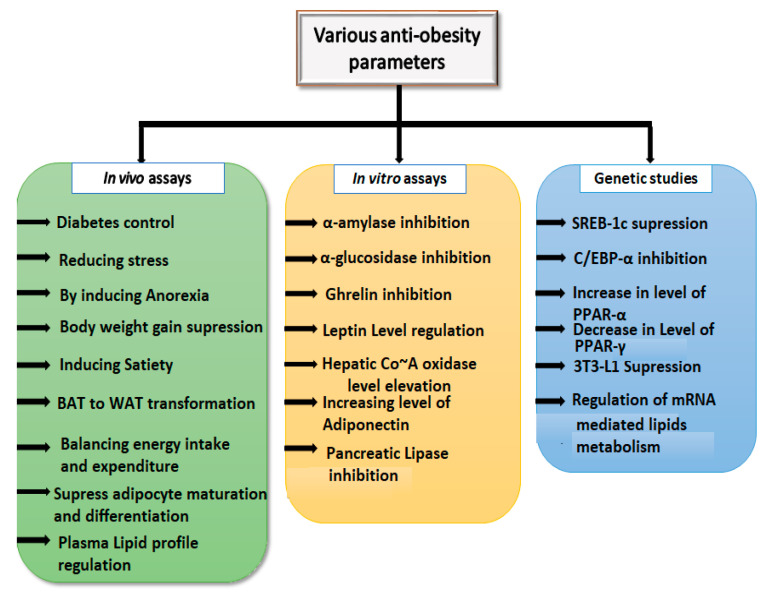
List of various parameters for assessing the anti-obesity efficacy of various natural products.

The natural products present in different plant resources alter the regulation of various enzymes and genetic factors through different acting mechanisms (Figure 2). These secondary metabolites inhibit carbohydrate and lipid metabolizing enzymes like α-amylase, α-glucosidases and different lipases present in the gastrointestinal tract. Amylases and glucosidases are the main key enzymes responsible for digesting the carbohydrates and result in release of glucose via glucose transporters [11]. An increase in the level of glucose above normal ultimately results in the release of insulin from pancreatic cells and initiation of three pathways (glycogenesis, glycolysis and de novo lipogenesis) to decrease glucose in the blood [12]. The synthesis of lipids and fatty acids from glucose and esterification of these lipids into triglycerides for storage in adipose tissue causes obesity [13]. On the other hand, lipases, mainly secreted from the different regions of the gastrointestinal tract, are mainly involved in the digestion of fatty acids, phospholipids and triglycerides and hydrolyze them into monoglycerides. These monoglycerides form chylomicrons and the micellar structure with sugars, lysophosphatidic acid and bile salts. This structure then passes into the enterocytes and subsequently results in the synthesis and storage of triglycerides in adipose tissue [14]. The inhibition of these enzymes, after treatment with plant products, ultimately results in the reduction of obesity. These secondary metabolites reduce obesity through modulation of different hormones such as leptin, ghrelin and insulin. Leptin is primarily secreted by white adipose tissue (WAT) [15] and regulates the “brain–gut axis” by activating its receptors in the central nervous system (CNS), subsequently reducing food intake and enhancing the calorie expenditure pathways [16]. Insulin is secreted from the pancreatic beta cells and transforms signals to the brain that result in a reduction in food intake over the long term and more rapid energy expenditure. Signals from both leptin and insulin communicate in a way to reduce the food and energy intake [17]. Plants with anti-obesity properties increase the level of both hormones. Adiponectin (an adipokine secreted from adipose tissue) increases hepatic insulin activity, increases fatty acid oxidation, and enhances glucose uptake in both skeletal muscle and the liver [16]. It mainly acts through the activation of adenosine monophosphate-activated protein kinase (AMPK) activity and AMPK impedes acetyl Co~A carboxylase activity and decreases the content of malonyl Co~A [18]. 

Ghrelin is also called the hunger hormone and inhibition of the secretion of ghrelin has an anti-obesity effect [19]. The process of adipogenesis and adipocyte differentiation can be interfered by regulating various transcriptional factors involved in different steps of these processes to manage obesity [20]. These transcriptional factors are proliferator-activated receptors (PPAR), sterol regulatory elementary binding proteins (SREBP) and CCAAT/enhancer binding proteins (C/EBP) [21]. Repression at the level of SREBP [22,23] and C/EBP [24] and enhancement of the PPAR level [25] are also strategies to manage obesity by different plant metabolites. Regulation of lipid metabolism at the level of synthesis and lipid degradation by different enzymes and hormones can reduce the obesity effect [26]. SREBP1a (sterol regulatory element binding protein 1a), SREBP2, low density lipoproteins (LDL), and receptors-3-hydroxy-3-methylglutaryl Co~A reductase mainly regulate the process of synthesis of cholesterol from acetyl Co~A [27]. SREBP-1c upregulates the transcription of the lipogenic enzymes stearoyl Co~A desaturase and fatty acid synthase (FAS) [28]. Activation of AMPK interferes with SREBP-1c and FAS and reduces the synthesis of cholesterol and fatty acids [29]. In a similar way, carnitine palmitoyl transferase 1A (CPT1A) decreases the concentration of hepatic triglycerides and increases the process of fatty acid oxidation [30]. Therefore, regulation of all of these factors imparts a beneficial effect in preventing obesity with the help of natural products that are secondary metabolites obtained from plants. Their role in treating obesity is described below. 

### 2.1. Polyphenols

**Figure 3:**
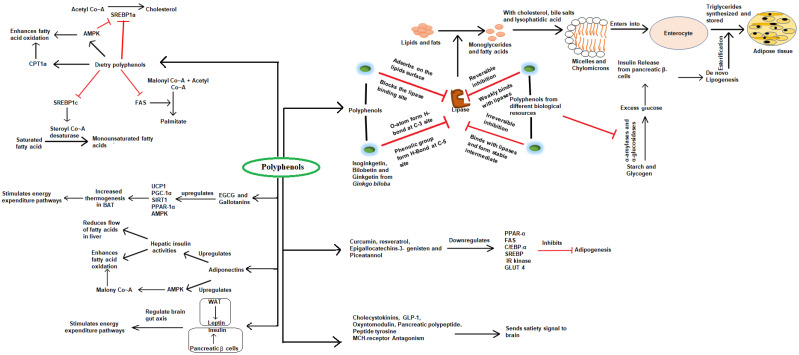
Diagram showing the different steps mainly regulated by polyphenols in managing obesity.

Polyphenols are phenolic compounds with at least one or more aromatic ring/s with a hydroxyl group and other functional groups like glycosides, methyl ethers and esters associated with its chemical structure [31]. On the basis of the number of aromatic rings, polyphenols can be categorized into, among others, tannins, stilbenes, phenolic acid, flavonoids, lignans, lignins and coumarins [32,33]. Among all the categories of phenolic compounds, flavonoids can be distinguished by the presence of two aromatic rings, connected by a 3-C bridge. Resveratrol, catechins, quercetin, procyanidins, epigallocatechins gallate, anthocyanins and procyanidins are gaining much interest due to their significant anti-obesity properties. Several studies have reported anti-obesity efficacy of different phenolic compounds in both animals and cell models. The polyphenols (extracted from *Vitis rotundifloia*) [34], ellagitannins, and proanthocyanidins extracted from raspberries and strawberries [35] are reported to inhibit pancreatic lipase with an IC_50_ value of 16.90 and 5 µg/mL, respectively. Gallic acid, epigallocatechin’s gallate (EGCG) and epigallocatechin are reported to inhibit the lipase activity with an IC_50_ value of 387.2, 273.3 and 39.2 µM, respectively [36]. Liu et al. [37] reported the inhibition of pancreatic lipase, α-amylase and α-glucosidases with an IC_50_ value of 1.86, 0.38 and 2.20 mg/mL, respectively, by phenolic compounds extracted from *Nelumbo nucifera.* Polyphenols extracted from *Citrus aurantium* [38] and *Coralluma fimbriate* [39] are reported to show appetite suppressive effects. MacLean and Luo [40] reported that Bushman’s Hat (*Hoodia Gordoni*) extracts increased ATP (adenosine triphosphate) in hypothalamic neurons and positively regulated hunger and food intake in rats. Epigallocatechins-3-gallate (EGCG) extracted from green tea reduced food intake by inhibition of ghrelin hormone and stimulation of adiponectin [41]. Stimulation of thermogenesis and energy consumption is also a significant way to decrease obesity. BAT is unique as it involves release of excess energy by the process of thermogenesis. An uncoupling protein (UCP1) regulates the process of thermogenesis in BAT by reducing the proton gradient and uncoupling ATP synthesis from oxidation [42]. UCP3 is another homologous protein to UCP1 and exerts its anti-obesity action by regulating the level of leptin, thyroid hormones and β-adrenergic agonists [43]. EGCG extracted from green tea is reported to induce thermogenesis and energy consumption [44]. Other compounds like quercetin, isoflavones, gallic acid, resveratrol, and curcumin induce thermogenic activity by modulating the signaling pathway of adenosine monophosphate protein kinase (AMPK), SIRT1 (sirtuin 1), proliferator activated receptor gamma coactivator 1-α (PGC-1α), and catechol O-methyl transferase, which are mainly involved in the regulation of transcription and physiology of adipose tissue (Figure 3). Curcumin, resveratrol, epigallocatechin-3-gallat, and genisten are able to inhibit the process of differentiation of adipocytes [45]. Carvacrol [46] and phenolic compounds from chokeberries [47] inhibited the process of adipocyte differentiation by modulating the level of PPAR-γ, C/EBP-α and SREBP-1c. Lipid and triglyceride accumulation is mainly responsible for excessive weight gain and obesity. The process of synthesis of cholesterol from acetyl Co~A is mainly regulated by SREBP1α, SREBP2, LDL receptors and 3-hydroxy methylglutaryl Co~A reductase [27]. AMPK stimulates fatty acid oxidation and reduces the synthesis of cholesterol by interfering with fatty acid synthase and SREBP-1c [29]. Polyphenols extracted from *Hibiscus sabdariffa* [48] and combined with the extract of *Lippia citridora* [49] exhibited anti-obesity efficacy.

### 2.2. Flavonoids 

**Figure 4:**
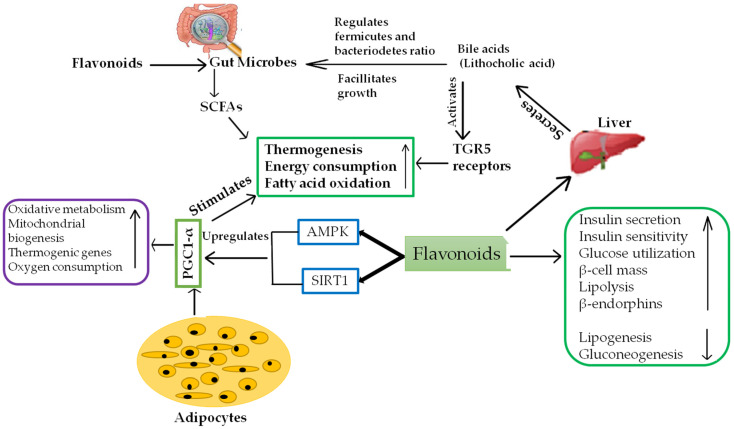
Diagram showing the primary mechanism that flavonoids have anti-obesity effects via different pathways.

Flavonoids, commonly present in a variety of plants, are mainly responsible for the flavor and colour of the vegetables and fruits [50]. The chemical structure of flavonoids mainly consists of a heterocyclic pyran ring and two aromatic rings associated with it, which forms a 15-C phenylpropanoid core. Flavonoids are grouped into six categories on the basis of the double bond present in the heterocyclic ring and its oxidation status. These groups are anthoxanthins (flavonols or catechins), anthocyanins (cyanin pigment), flavonones (narigenin and herpertin), flavones (luteolin, apigenin), isoflavones (genistein, flavin) and chalcones (butein, xanthoangelol) [51]. These different groups of flavonoids show demarcated anti-obesity properties with different modes of action. Flavonoids inhibit weight gain by reducing food intake and increasing the feeling of satiety. Flavonoids extracted from the spinach leaf and the combination of flavonoids and procyanidin [52] had a significant effect in treating overweight by reducing the cravings for food and increasing satiety. WAT is specialized in storage of excess energy in the form of triglycerides [53] and BAT is mostly specialized for high metabolism and energy expenditure. It is predominantly present in the suprarenal, spinal and supraclavicular regions [54]. Both adipocytes express UCP-1 protein, which is mainly responsible for thermogenesis and energy consumption. Thermogenesis is mainly regulated by various mechanisms and improves the activity of the sympathetic nervous system, which results in the secretion of norepinephrine, which ultimately results in energy consumption and reduction of fat accumulation [55]. PGC1-α is the main transcription factor that regulates the process of thermogenesis [56]. AMPK and SIRT1 are mainly increased by flavonoids and are the factors mainly responsible for the expression of PGC1α. AMPK/PGC1α signaling is primarily responsible for browning of adipose tissue and thermogenesis [57]. The gastrointestinal tract comprises a diverse bacterial population, including Actinobacteria, Firmicutes, Proteobacteria and Bacteroidetes [58], and out of which 1.9% of total flora are heritable and more than 20% of the diversity mainly depends on environmental factors such as dietary habits [59]. Imbalance in the diversity of the gut microflora may cause endotoxic accumulation in the circulatory system, which in turn may induce chronic inflammation and obesity [60]. Short chain fatty acids (SCFAs) are produced after bacterial fermentation of some indigestible biochemicals such as polyphenols, polysaccharides and protein, which are mainly involved in energy expenditure, oxidation of fatty acids, regulation of sympathetic activity and intestinal gluconeogenesis [61]. Bile acid produced from cholesterol in the liver can be metabolized by intestinal bacteria and bile acid also regulates the composition of microbes and facilitates their growth [62]. The aforementioned factors reveal the role of flavonoids in obesity regulation (Figure 4).

### 2.3. Diterpenoids 

**Figure 5:**
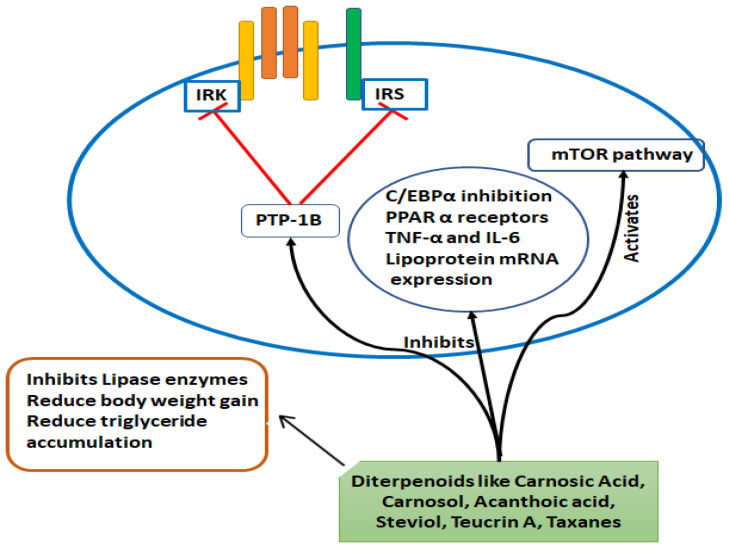
The anti-obesity mechanisms of diterpenoids.

Diterpenoids are chemical compounds that contain two terpene units, with each having four isoprene units with the molecular formula C_20_H_32_ [63]. The anti-obesity mechanism of diterpenoids is depicted in Figure 5. Diterpenoids have several therapeutic effects, such as anti-obesity effects from taxanes from Taxus [64] carnosic acid, and steviol and its derivatives [65]. Diterpenoids and their derivatives showed anti-obesity properties through different strategies. Teucrin A isolated from *Teucrin chaemodrys* and Carnosic acid isolated from *Rosmarinus officinalis* are reported to reduce body weight in obese Sprague Dawley rats [66] and mice [67], respectively. Secondly, PTP-1B (protein tyrosine phosphatase 1B) was shown to mainly have adverse effects on leptin transduction and insulin signaling, and the inhibition of this enzyme is reported to speed up the insulin signaling pathways and in turn have positive effects in treating obesity [68]. Acanthoic acid, isolated from *Acanthopanax koreanum* [69] and Ent-16βH, 17-isobutryloxy-kauran-19-oic acid and Ent-16βH, 18-isobutryloxy-kauran-19-oic acid isolated from *Siegesbeckia glabrescens* [70], and Hueafuranoid A isolated from *Huea* sp. are reported to inhibit PTP-1B activity in a dose dependent manner (IC_50_ = 30 µg/mL and 13.9 µM, respectively). Diterpenoids also showed lipase inhibitory effects as they inhibited the activity of pancreatic lipase and triglyceride accumulation. Carnosic acid (CA) (isolated from *R. officinalis*) and carnasol (isolated from *Salvia officinalis* [71] showed lipase inhibitory activity and modulated body weight gain [3] and lipoprotein-lipase mRNA expression in mouse adipose tissue [72]. Diterpenoids showed anti-obesity effects by inhibiting adipocyte differentiation. Carnosic acid [72,73] and 14-deoxy-11,12- didehydroandrographolide isolated from *Andrographis paniculata* [74] are reported to interfere with mitotic clonal expansion, block the expression C/EBPα and PPAR-α, reduce lipoprotein mRNA expression via tumor necrosis factor (TNF-α) and interleukin-6, alter the ratio of different C/EBP-β proteins, and activate the mTOR pathways. In a study on the geranylgeraniol (alcoholic derivatives of diterpenoids), which are mostly found in some herbs and fruits, it was reported that they activate human PPARα and PPARγ in CV1 cells and regulate the expression of target genes mainly responsible for lipid metabolism in 3T3-L1 cells and Hep G2 [75].

## 3. Anti-Obesity Effects of Different Plant Resources 

Different plant extracts with different anti-obesity effects are described in Table 1. The plant extracts of *Curcuma longa* [76], *Camellia sinensis* [77], *Cosmos cadatus* [78], *Morniga olifera* [79] and *Elateriospermum tapos* [80] are reported to have anti-obesity efficacy and among these plants *Curcuma longa* is reported to have the highest anti-obesity potential. It decreased body weight and adipose tissue weight, and more importantly, regulated the expression of transcription factors like PPAR-γ (71 %) and C/EBP-α (38.83%). Fatty acyl synthase (FAS) (66.03%) and acetyl Co~A carboxylase (ACC) (35%) decreased and that of AMPK (12.93%), CPT1 (88%) and adiponectin (49.01%) increased after the treatment. Other plants also regulated the serum lipid profiles, including TG, TC, ALT, AST and ALP concentration and expression of the transcription factors responsible for lipid metabolism. C57BL/6J mice were also implemented as an experimental model for *Nelumbo nucifera* [81], chlorogenic acid [82], *Capsicum annuum* [83], *Cudrania tricuspidate* [84], *Morus alba*, *Ilex paraguariensis*, *Rosmarinus officinalis*, *Moringa officinalis* [85,86], *Ecklonia cava* [47], *Cirsium cetidens* [87], *Gymnema sylvestre* [88] and *Ishige okamurae* [89], and among all these plants, the mixed extracts of *Morus alba*, *Ilex paraguariensis and Rosmarinus officinalis* showed the maximum anti-obesity effect as it decreased the body weight and relative weight of other body organs up to 98% and regulated the serum lipid profile by decreasing the TC (18.6%), LDL-c (59%), ALT (60.1%), AST (35.2%), insulin (75.9%) and leptin (46.8%). These results are maximum among the plants described above in Table 1 on C57BL/6J mice. The secondary metabolites from all the plants also reduced the expression of those transcription factors (PPAR-γ, C/EBPα, SREBP-1c) that are mainly responsible for the accumulation of lipids in adipose tissue and promoted the expression of those factors (ACC, PPARα, AMPK and adiponectins).

Among the anti-obesity effects of *Juniperus communis* [104], *Ramulus mori* [111], *Vibrunum opulus* [106], *Paullinia cupana* [96], *Peucedanum japonicum* [23], *Aster yomena* [97], *Prunus salicina* [98], *Eclipta alba* [99], and *Polygonum cuspidatum* [92] evaluated on 3T3-L1 cells, Eclipta alba showed the highest activity as it decreased the expression of PPARα (1.9 fold), C/EBPα (1.8 fold), FAS (1.4 fold) and FABP4 (1.8 fold) and also decreased the concentration of lipids in terms of cholesterol and triglycerides in these adipocytes. The above-described plants regulated the transcription factors and lipid accumulation in 3T3-L1 adipocytes. Other biochemical parameters like inhibition of digesting enzymes were also reported in the studies on *Solenostema argel*, *Coralluma quadrangular*, *Hibiscus sabdariffa* [103], *Helichrysum sanguineum* [110], *Vitis vinifera*, and *Rhus coriaria* [100]. *Solenostema argel* showed the maximum inhibition of pancreatic lipase (97.02 ± 1.4%), α-amylase (69.32 ± 1.14%) and α-glucosidase (89.08 ± 1.1%).

## 4. Nanotechnology Associated with Anti-Obesity Effects 

Nanotechnology and nanoencapsulation of secondary metabolites is an emerging and more beneficial strategy for the treatment of obesity with amplified efficiency and minimized side effects [117]. Various types of phytochemicals such as flavonoids, terpenoids, polyphenols, glycosides and tannins have been reported as promising agents in treating obesity but their low target specificity, low aqueous solubility, stability and toxicity at high dose put some restrictions on their clinical use. These biomolecules are mainly responsible for the reduction of metal ions from their metallic precursor and green synthesis of metallic nanoparticles. These limitations can be overcome by using metallic nanoparticles and nanoencapsulated phytochemicals, as they increase their target specificity, bioavailability, and solubility, and more importantly, prevent them from pre-term degradation [10]. They also have a high surface to volume ratio and tunable surface chemistry. Table 2 shows the list of some nanoparticles synthesized from the biological material and some nanoencapsulated biological molecules with their anti-obesity effects on obese animal models and/or cell lines.

The anti-obesity effect of the nanocellulose compound isolated from *Vitis vinifera* was reported by Abdelbaky et al. [118] on a rat model. They reported the regulation of body weight, organ weight, blood serum lipid profile and food intake after induction of cellulose NPs in their diet. The nanoparticles isolated from red grape seed, especially cellulose nanocrystals (CNC), had a significant positive effect on obesity and hyperlipidemia compared to grape seeds powder, while the chemical constituents of the crude leaf extract of *Vitis vinifera* [100] inhibited pancreatic lipase, affecting lipid metabolism and consequently obesity. In a similar way, the gold nanoparticles from *Salacia chinensis* [119], *Smilax glabra* [120], *Poria cocos* [122], *Dendropanax morbifera* [130] and chitosan NPs [121] were reported to show anti-obesity efficacy by regulating the serum lipid profile and the level of hormones related to lipid metabolism and regulation of the transcriptional factors mainly responsible for the metabolism of lipid digestion and lipid accumulation. Nanoencapsulation increased the delivery of the specific molecule/secondary metabolite to the specific target with much greater efficacy and stability. Nanoencapsulation of quercetin with a succinyl chitosan alginate shell [124] and PLGA [128] in rat models revealed that the serum lipid profile and glucose level were regulated after the induction of these nanoencapsulated structures. On the other hand, quercetin, being one of the chemical constituents in extracts of different plant parts—flowers of *Capsicum annuum* [94], plums of *Prunus salicina* [98], leaves of *Vitis vinifera* [100], fruits of *Rhus coriaria* [100], leaves of *Cosmos cadatus* Kunth [78], fruits of *Malus prunifolia* [102], fruits of *Malus huphenesis* [102], *Hibiscus sabdariffa* [103], *Solenostemma argel* [103], and *Vibrunum opulus* [105], twigs of *Ranulus mori* [111], leaves of *Nelumbo nucifera* [81], soybean embryo and enzymatically modified Isoquercetin [115]—has contributed to obesity management as mentioned above.

EGCG is a secondary metabolite mainly present in green tea and many other plant products and its encapsulation with Soy PC, α-tocopherol and Kolliphor HS15 [126] and Kodia-PC and α-tocopherol [125] was reported in C57BL/6J mice and Human THP1 cells, respectively. In mice, it regulated the serum lipid profile and decreased the surface lesion of aortic arches and inflammatory factors, and in monocytes, it mainly decreased the expression of the transcription factors responsible for lipid accumulation and in turn decreased the cholesterol level in these monocytes.

Similarly, the anti-obesity studies on *Curcuma longa* [76] and the nanoencapsulated curcumin [72,127] molecule showed that the curcumin molecule capsulated with a PLA-PEG molecule enhanced the anti-obesity effect in terms of modification of the serum lipid profile (*p* ≤ 0.01) and modulation of transcription factors (*p* ≤ 0.01) compared to native curcumin molecules [76].

Resveratrol and oxy resveratrol are chemical ingredients of twigs of *Ranulus mori* [111], root extract of *Polygonum cuspidatum* [92], and a nanocellulose compound isolated from *Vitis vinifera* [118]. Trans resveratrol-encapsulated NPs [131] regulated the expression of signal pathways, lipid parameters and conversion of WAT to BAT related to obesity management. These chemicals acted individually (NPs) and/or in combination (crude/extract) in obesity management. Resveratrol was encapsulated with PLGA by Wan et al. [129], who used HepG2 cells to evaluate the anti-obesity efficacy of these nanoencapsulated structures. They found a significant decrease in the lipid accumulation, hepatocellular differentiation and triglyceride accumulation in these hepatocytes.

More studies are required, which will certainly open new avenues and add further to existing knowledge to establish the contribution of the constituent chemical/s (nanoencapsulated or constitutent of extract), individually or synergistically, that may act as efficacious nanomedicines assisting in obesity management, specifically acting through its regulatory parameter/s.

## 5. Conclusions

The human population has witnessed the health benefits of natural compounds due to the presence of secondary metabolites in medicinal plants. The secondary metabolites have modulating effects in various disorders. The natural compounds have less side effects. Obesity is an ordinary underrated health disorder but is now considered a serious public health issue globally, which leads to the emergence of other chronic health disorders. This review summarizes the natural products in plant extract(s)/isolated or purified compound(s) and the nanomedicine-enhanced effect of these natural compounds to reduce the comorbidities related to obesity. The nanomedicine-based approach showed curative effects for obesity management by their gene target specific activity and this drug delivery system opens up a new horizon to improve the ameliorative efficiency of these natural compounds. To cite an example, the nanoencapsulated curcumin (PLA-PEG) molecule showed significantly enhanced anti-obesity effects compared to the native curcumin molecule. The nanoparticles and nanoencapsulation treatment enhanced the anti-obesity efficacy. This article also aimed to explain the mechanism of these secondary metabolites in regulating the obesity parameters by modulating the gene/transcriptional factors amenable for adipogenesis, adipocyte differentiation, energy and lipid metabolism, and gut microbiota. Future perspective studies of such natural molecules in terms of drug specificity, efficacy and ethical issues/trials need to be conducted for their specific validation in the interest of better human health. 

## Figures and Tables

**Table 1:** List of different plants with anti-obesity effects with the list of secondary metabolites responsible for this biological activity.

Sr. No.	Plant	Secondary Metabolite	Experimental Model	Important Findings	References
1.	Rhizome of *Curcuma longa* (Zingiberaceae)	Curcumin	Sprague-Dawley rats	Body weight gain, perirenal and epididymal adipose tissue weight decreased (*p* ≤ 0.05) PPAR-γ (71%), C/EBPα (38.83%), FAS (66.03%), ACC (35%) level decreased AMPK (12.93%), Adiponectin (49.01%) an CPT1 (88%) increased TG and LDL level decreased (*p* ≤ 0.05)	[76]
2.	Leaves of *Gymenma sylvestra* (Apocynaceae)	Deacyl gymnemic acid	C57BL/6J mice	Body weight gain, epididymal fat decreased, lowers food and energy efficiency ratio TC (30.44%), TG (18.53%), LDL (33.69%), Leptin (35.52%) decreased and HDL (20.26%) increased	[88]
3.	Root, root cortices and root corks of *Zicao* (Boraginaceae)	Acetylshikonin	Sprague-Dawley rats and 3T3-L1 adipocytes	Food efficiency ratio decreased to 10.0 ± 0.14%, Body weight gain, Epididymal adipose tissue weight (18.7 ± 0.23%), adipocytes size (*p* ≤ 0.001) at 100 mg/kg FFA (35.5%) and serum TG level (39.9%) reduced at 900 mg/kg Lipolysis (0.85 fold), glycerol secretion (0.50 folds) increased, PPAR-γ and C/EBP reduced at 1.5 µM (*p* ≤ 0.05), Adipocytes differentiation decreased	[90]
4.	Rhizomes of *Dioscorea oppositifolia* (Dioscoreaceae)	(3*R*, 5*R*)-3,5 dihydroxy-1,7-bis (4-hydroxyphenyl)-3,5-heptanediol and 3,5-dimethoxy-2,7-phenanthrenediol	Mice	Body weight gain (45.2%) and adipose tissue weight (22.5%) decreased, TG (18.8%), TC (15.4%), hepatic TG (48.1%) and Hepatic TC (46.9%) decreased, HDL-c (34.2%) increased at 100 mg/kg dose	[91]
5.	Leaves of *Peucedanum japonicum thumb* (Umbelliferae)	*Cis*-3′,4′-diisovalerylkhellactone (cDIVK)	3T3-L1 adipocytes	TG (65%), adipocyte differentiation (80%) decreased AMPK phosphorylation increased (103%) C/EBPα, PPARγ and SREB-1c (*p* ≤ 0.05) Inhibition of α-glucosidase activity at 50 µM concentration	[92]
6.	Seeds of *Capsicum annuum* (Solanaceae)	Capsicoside G-rich protein (CRP-13.35%)	C57BL/6J mice	Body weight, food consumption and food efficiency decreased (*p* ≤ 0.05) Adipocyte and epididymal adipose tissue weight decreased TG, TC, AST, ALT level decreased (*p* ≤ 0.05) C/EBPα, PPARγ, SREBP1c, FAS decreased (*p* ≤ 0.05)	[83]
7.	Tubers of *Solanum tuberosum* (Solanaceae)	Proteinase inhibitors	Mice	Body weight gain (18.5%), food consumption (39.93%), epididymal fat (52.40%), TC (19.65%), FFA (33.33%), TG (28.60%), leptin (7.35%) and adipocyte differentiation decreased at 300 mg/kg Adiponectin increased (33.62%)	[93]
8.	Fruit of *Diospyros kaki* (Ebenaceae) and peel of *Citrus unshui* (Rutaceae)	Flavonoids and phenolic compounds	Mice	Body weight gain decreased (16.62%) visceral fat weight (12.52%) decreased and pancreatic lipase inhibited (about 50%) at 500 µg/mL TC, TG, LDL decreased and HDL increased (*p* ≤ 0.05) at 50 mg/kg	[70]
9.	Leaves of *Cudrania tricuspidata* (Moraceae and *Lonicera caerulea* (Caprifoliaceae) and whole soyabean plant of *Glycine hispida* (Fabaceae)	Anthocyanins, flavonoids, phenolic compounds, anthoxanthines, prenylated flavonoids, isoflavone and soyasaponins	C57BL/6J mice	Subcutaneous fat (37.10%), Adipose area (39.85%), Adipose tissue (25.22%), TC (10.40%), TG (53.80%), LDL (14.56%), ALT (38.46%), Leptin (66.36% decreased)	[84]
10.	Flower of *Capsicum annuum* (Solanaceae)	Phenolic compound, flavonoids, chlorogenic acid and quercetin	Macrophages RAW-264.7	Pancreatic lipase inhibited (IC_50_ = 3.54 ± 0.18) to 82.54% at 10 mg/mL Inhibition of lipids peroxidation (IC_50_ = 27.61 ± 2.25 µg/mL) after 30 min and (IC_50_ = 41.69 ± 1.13 µg/mL) after 60 min of incubation	[94]
11.	Leaves of *Morus alba* (Moraceae), root bark of *Yerba mate* (Aquifoliaceae) and stem bark of *Magnolia officinalis* (Magnoliaceae)	Xanthines, purines, alkaloids, flavonoids, polyphenols	C57BL/6J mice	Body weight gain (98.6%) and relative weight of body organs (*p* ≤ 0.001) decreased, TC (18.6%), LDL (59%), ALT (60.1%), AST (35.2%), insulin (75.9%), leptin (46.8%) decreased and ghrelin hormones increased (4.2 folds) at 350 mg/kg	[85]
12.	Aerial part of *Caralluma fimbriata* (Asclepiadeceae)	Organic acid, amino acid, carbohydrates, pregnane and trigonelline	Rat	Decreased the hypothalamic level of NPY, ORX peptide Insulin level (29.52%) increased AST (22.61%), ALT (12.12%) ALP 22.07%) and Lipase (7.5%) decreased	[95]
13.	*Paullinia cupana* (Sapindaceae)	Phenolics, flavonoids, caffeine, theobranium, theophylline, tannins, saponins, catechins, epicatechins, proanthocyanins	3T3-L1 adipocytes	TG accumulation decreased at 300 µg/mL Upregulated the expression of anti-adipogenic genes FOXO1, Gata3, Dlk1, Wnt1, Wnt3a, Wnt10b Decreased the expression of adipogenic markers PPAR-γ and CEBP-α and SREBP-1	[96]
14.	Leaves of *Cirsium setidens* (Asteraceae)	Pectolinarin	3T3-L1 adipocytes and C57BL/6J mice	Lipid accumulation (56%) and body weight gain (18.46%) decreased at 200 µg/mL, AST (3.1%), ALT (4.15%), TC (8.73%), TG (22.45%) decreased, Expression of PPARα, C/EBPα, C/EBP-δ and FABP4 downregulated at 200 µg/mL	[87]
15.	Root bark of *Morus alba* (Moraceae), leaves of *Ilex paraguariensis* (Aquifoliaceae) and leaves of *Rosmarinus officinalis* (Lamiaceae)	Bioflavonoids, morusin, Kuwanon G, carnosol, carnosic acid, caffeine, polyphenols, xanthines, purines and alkaloids	C57BL/6J mice	Body weight (30.2%), TC (21.1%), TG (44.6%), LDL (38.2%) decreased at 800 mg/kg, Level of leptin (16.4%) and ghrelin (2.1 folds) decreased and of insulin (65.5%) increased	[86]
16.	Leaves of *Aster yomena* (Asteraceae)	Phenolics compounds and terpenoids	3T3-L1 adipocytes	Expression of PPARγ, C/EBPα, C/EBPβ and SREBP-1c decreases significantly in a conc. dependent manner (*p* ≤ 0.05), Phosphorylation of AMPK increased, Lipids accumulation reduced	[97]
17.	Leaves, stem and roots of *Ecklonia cava* (Lessoniaceae)	Phlorotannins, fucodiphloroethal	C57BL/6N Mice	Body weight (0.82 fold), liver weight (2.14 ±0.31 to 0.97 ± 0.14 g), epididymal adipose tissue (0.29 ± 0.02 to 0.20 ± 0.02), perirenal adipose tissue (0.63 ± 0.08 to 0.33 ± 0.05 g), TC (83.6 ± 6.10 to 59.9 ± 16.6 mg/dL), TG (57.9 ± 12.16 to 38.0 ± 5.05 mg/dL) decreased at 150 mg/kg HDL-c increased (38.4 ± 3.87 to 61.2 ± 7.39 mg/dL), level of SREBP-1c, FABP, FAS decreased and AMPK phosphorylation increased (*p* ≤ 0.05)	[47]
18.	Plums of *Prunus salicina* (Rosaceae)	Phenolic compounds, flavonoids, chlorogenic acid, hydroxycinnamic acid, epicatechin, quercetin	3T3-L1 adipocytes	Adipocytes differentiation decreased, PPAR-γ, C/EBP-α, β-actin, decreased and AMPK phosphorylation increased (*p* ≤ 0.05)	[98]
19.	Whole plant of *Eclipta alba* (Asteraceae)	Demethylwedelolactone derivative, isodemethylwedelolactane, apigenin, isoechinmocystic acid-3-*O*-glycoside, Echinocystic acid	3T3-L1 adipocytes	Expression of PPARα (1.9 folds), C/EBPα (1.8 folds), FAS (1.4 folds), FABP4 (1.8 folds) decreased TC, TG and LDL decreased (*p* ≤ 0.05)	[99]
20	Leaves of *Vitis vinifera* (Vitaceae)	Phenolic compounds, phenolic acid, flavonoids-trans caffeoyl tartaric acid, myricetin-3-*O*-glycoside, quercetin-3-*O* glucoside, kaempferol-3-*O*-glucoside and isorhamnetin	Enzyme/s	Pancreatic lipase enzyme inhibited with IC_50_ 14.1 ± 1.9 µg/mL	[100]
21.	Fruits of *Rhus coriaria* (Anacardiaceae)	Caffeoylquinic acid, quercetin, rhamnetin, myricetin, kaempferol, gallic acid, methyl gallate, *m*-digallic acid and amenthoflavone	Enzyme/s	Pancreatic lipase enzyme inhibited with IC_50_ 19.95 ± 1 µg/mL	[100]
22.	Organic extract of *Origanum dayi*	Volatile oils, linalyl acetate terpinen-4-ol, α-terpineol, (*E*)-sabinene hydrate acetate, 1,8-cineole, (*E*)-sabinene hydrate	Enzyme/s	Pancreatic lipase enzyme inhibited with IC_50_ 26.9 ± 2.80 µg/mL	[100]
23.	Leaves of *Bacillus* fermented *Camellia sinensis* (Theaceae) green tea	Catechins, caffeine, gallocatechins, EGCG,	Sprague-Dawley rats	Inhibition of pancreatic lipase with IC_50_ of 0.48 mg/mL Post prandial lipaemia reduced Expression of PPAR-α (0.2 folds), SREBP-1c (0.7 folds), ACC (1.7 folds), FAS (1.7 folds) decreased Acetyl Co~A and CPT1 increased	[77]
24.	Leaves of *Cosmos cadatus* Kunth (Asteraceae)	Catechins, quercetin, rutin, kaempferol, chlorogenic acid	Sprague-Dawley rats	Body weight gain (60%) and visceral fat decreased at 350 mg/ kg Ghrelin level and leptin level (45.38%) decreased AST (158.90 ± 48.09 to 93.50 ± 11.25 mg/dL), ALT (45.95 ± 7.51 to 33.25 ± 6.80 mg/dL) and ALP (94.00 ± 11.17 to 79.75 ± 7.59 mg/dL) decreased at 175 mg/kg	[78]
25.	Bark of *Oroxylum indicum* (Bignoniaceae)	Oroxylin A, Chrysin and Baicalein	3T3-L1 adipocytes and Enzyme/s	Inhibition of pancreatic lipase (IC_50_ = 78.03 ± 1.67 µg/mL) and lipid accumulation decreased (IC_50_ = 70.13 ± 2.27 µg/mL) Downregulation of PPAR-γ, C/EBP-α	[101]
26.	Fruits of *Malus prunifolia* (Rosaceae)	Citric acid, *p*-coumaric acid, hyperoside, myricetin, naringenin, quercetin. Kaempferol, gentiopicroside, 8-epiloganic acid	Mice	Regulated the serum lipid parameters, TC (*p* ≤ 0.01), LDL-C (*p* ≤ 0.01) level decreased and HDL-C (*p* ≤ 0.05) increased	[102]
27.	Fruits of *Malus huphenesis* (Rosaceae)	*p*-coumaric acid, 8-epiloganic acid, hyperoside, myricetin, naringenin, Kaempferol, quercetin, gentiopicroside,	Mice	Regulated the serum lipid parameters, TC (*p* ≤ 0.05), LDL-C (*p* ≤ 0.05) level decreased and HDL-C increased	[102]
28.	*Caralluma quadrangula* (Apocyanaceae)	Phenolics, flavonoids, tannins and steroidal contents	Enzyme/s	Pancreatic lipase (62.56 ± 0.43%), α-amylase (54.31 ± 0.58%) and α-glucosidase (47.11 ± 1.3%) got inhibited	[103]
29.	*Hibiscus sabdariffa* (Malvaceae)	Delphinidin-3-sambubioside, cyanidin-3-diglucoside, Delphinidin, Luteolin, quercetin, gossypitrin, chlorogenic acid and protocatechuic acid, ellagic acid, *p*-coumaric acid, ferulic acid and caffeic acid	Enzyme/s	Pancreatic lipase (67.56 ± 3.63%), α-amylase (64.75 ± 1.2%) and α-glucosidase (64.25 ± 1.7%) got inhibited	[103]
30	*Solenostemma argel* (Apocyanaceae)	β-sitosterol, β-sitosterol glucoside, stemmoside C, Kaempferol-3-*O*-neohesperidoside, kaempferol-3-*O*-glucoside, quercetin-3-*O*-neohesperidoside,	Enzyme/s	Pancreatic lipase (97.02 ± 1.4%), α-amylase (69.32 ± 1.14%) and α-glucosidase (89.08 ± 1.1%) got inhibited	[103]
31.	Leaves of *Juniperus communis* (Cupressaceae)	Volatile oils, flavonoids and phenolic compounds	3T3-L1 adipocytes	α-amylase (97.5%) inhibited with IC_50_ 20.93 ± 1.067 µg/mL and α-glucosidase (98.4%) with IC_50_ 152.93 ± 1.067 µg/mL Lipase (97.5%) with IC_50_ 69.93 ± 1.067 at 1000 µg/mL Adipogenesis (67%) at lipids accumulation (69%) inhibited at 160 µg/mL	[104]
32.	Fruits of *Vibrunum opulus* (Adoxaceae)	Anthocyanidins, phenolics and flavonoids, quercetin, caffeic acid, dicaffeolquinic acid, neochlorogenic acid, catechin and rutin	3T3-L1 adipocytes	Pancreatic lipase (60%) and lipids accumulation (22%) inhibited at 75 µg/mL Expression of PPAR-γ and leptin decreased (*p* ≤ 0.001)	[105]
33.	Powder of plant *Spirrulina platensis* (Phormidiaceae)	Phospholipids, polyunsaturated fatty acids, provitamins, minerals, proteins and polysaccharides	Rat	Body weight, liver weight, BMI, hepatosomatic index decreased (*p* ≤ 0.05) ALT (134.23 ± 3.56 to 88.79 ± 4.79 U/L), AST (210.50 ± 9.97 to 184.00 ± 9.97 U/L), ALP (157.01 ± 4.43 to 98.27 ± 5.45 U/L) and leptin (0.38 ± 0.02 to 0.26 ± 0.01 ng/mL) decreased PGC-1α increased, FAS and PPAR-γ and decreased (*p* ≤ 0.05)	[106]
34.	Beans of *Coffea arabica* (Rubiaceae)	*p*-coumaric acid, quinic acid, chlorogenic acid, caffeine, and caffeic acid	Rat	Body weight, liver weight, BMI, hepatosomatic index decreased (*p* ≤ 0.05) ALT (134.23 ± 3.56 to 93.48 ± 5.07 U/L), AST (210.50 ± 9.97 to 193.69 ± 9.55 U/L), ALP (157.01 ± 4.43 to 84.12 ± 2.65 U/L) and leptin (0.38 ± 0.02 to 0.31 ± 0.004 ng/mL) decreased PGC-1α increased, FAS and PPAR-γ and decreased (*p* ≤ 0.05)	[106]
35.	Mixture of the *Spirrulina platensis* and *Coffea arabica*	Chlorogenic acid and unsaturated fatty acids like oleic acid, linolenic acid and linoleic acid	Rat	Body weight, liver weight, BMI, hepatosomatic index decreased (*p* ≤ 0.05) ALT (134.23 ± 3.56 to 73.37 ± 5.77 U/L), AST (210.50 ± 9.97 to 142.84 ± 9.5 U/L), ALP (157.01 ± 4.43 to 66.77 ± 2.79 U/L) and leptin (0.38 ± 0.02 to 0.17 ± 0.003 ng/mL) decreased PGC-1α increased, FAS and PPAR-γ and decreased (*p* ≤ 0.05)	[106]
36.	Pentacyclic triterpenoids isolated from the Styrax	Betulinic acid, epibetulinic acid, oleanic acid, oleanonic acid, betulonic acid, corosolic acid, maslinic acid, cinnamyl cinnamate, 3-phenylpropyl cinnamate	Enzyme/s	Pancreatic lipase inhibited by Oleanonic acid with IC_50_-0.49 µM, betulonic acid with IC_50_-1.48 µM, oleanolic acid with IC_50_-3.53 µM, maslinic acid with IC_50_-6.06 µM and corosolic acid with IC_50_-6.35 µM	[107]
37.	*Pogostemum cablin* (Lamiaceae)	Sesquiterpene alcohol (Patchouli alcohol)	3T3-L1 adipocytes and C57BL/6J mice	TG accumulation (71.4%), PPAR-γ (58.8%) and C/EBPα (77.3%) decreased at 100 µM, β-catenin increased (36.6%) Body weight, epididymal, retroperitoneal, and brown fat weight decreased (*p* ≤ 0.05)	[108]
38.	Seeds oil of *Moringa olifera* (Moringaceae)	Tannins, flavonoids, terpenoids, glycosides and saponins	Sprague-Dawley rats	Body weight (226.33 ± 8.53 to 201.17 ± 15.82g), Kidney weight (0.30 ± 0.01 to 0.25 ± 0.03 g) and epididymal tissue (0.97 ± 0.05 to 0.90 ± 0.09 g) decreased TC, ALT, AST, Creatinine and uric acid declined significantly (*p* ≤ 0.05)	[79]
39.	Seeds of *Theobroma cocao* (Malvaceae)	Proteins isolated; Vicillin and albumin	Male wistar rats	Pancreatic lipase inhibited with IC_50_ value 1.4 mg /mL Body weight (301.6 ± 4.60 to 278.5 ± 6.50 g) decreased Total lipids, TG, TC decreased significantly (*p* ≤ 0.05) at 150 mg/kg	[109]
40.	Chlorogenic acid	Phenolic acid	C57BL/6J mice	ALT, AST, ALP decreased significantly (*p* ≤ 0.001) Improves the gut microbiota and contributes to 66 total bacterial species out of 117 Body weight gain decreased (15%) (*p* ≤ 0.05) BAT activity increased	[82]
41.	Shell extract of *Elateriospermum tapos* (Euphorbiaceae)	3′,4′,5′-trimethoxyflavone, acetyllysine, 7-methoxy chromone, undulatone, aldosterone 18-glucouronide	Sprague-Dawley rats	Body weight (350.0 ± 15.0 to 288.0 ± 42.8 g), calories intake (9341.9 ± 781.52 to 7971.6 ± 945.46 KJ), Liver, viscera fat and gonadal fat decreased (*p* ≤ 0.05), TC (1.63 ± 0.33 to 1.48 ± 0.16 mMol/L), LDL-C (1.04 ± 0.16 to 0.86 ± 0.20 mMol/L), TG (1.77 ± 0.84 to 1.32 ± 0.23 mMol/L) lipoprotein lipase activity (1.77 ± 0.84 to 1.32 ± 0.23) decreased and HDL-C increased (0.35 ± 0.05 to 0.41 ± 0.08)	[80]
42.	Aerial part of *Helichrysum sanguineum* (Asteraceae)	Cardiac glycosides, phenols, volatile oils, tannins, Steroids, reducing sugar and flavonoids	Enzyme/s	α-amylase and pancreatic lipase got inhibited with IC_50_ value of 28.18 ± 1.04 to 63.09 ± 0.3 µg/mL	[110]
43.	Twig of *Ramulus mori* (Lamiaceae)	Quercetin 3-β-glycosides, quercetin Mulberroside, oxyresveratrol and resveratrol	3T3-L1 adipocytes and Mice	Body weight (27.3%), liver weight (17.5%) and epididymal tissue weight (19.5%) decreased at 100 mg/kg TC (211.5 ± 16.4 to 165.8 ± 7.0 mg/dL), TG (97.7 ± 8.5 to 65.0 ± 14.2 mg/dL), LDL-C (46.3 ± 19.1 to 24.5 ± 13.9 mg/dL) decreased and HDL-C (46.3 ± 19.1 to 24.5 ±13.9 mg/dL) increased PPAR-γ (0.96 fold), C/EBPα (0.92 fold), SREBP (0.92 fold) decreased and ACC (0.78 fold), FAS (0.96 fold) and SCD (0.84 fold) increased at 40 µg/mL	[111]
44.	Leaves of *Nelumbo nucifera* (Nelumbonaceae)	Kaempferitrin, hyperoside, astragalin, phloretin and quercetin	C57BL/6J mice	ALT, AST, ALP, TG, TC and HDL-C decreased (*p* ≤ 0.05), HDL-C increased (*p* ≤ 0.05) Liver index (4.17 ± 0.22 to 3.93 ± 0.21) and epididymal fat index (1.55 ± 0.28 to 1.14 ± 0.21) decreased, Expression of PPAR-γ and C/EBPα decreased and PPAR-α, CPT1 and CYP7A1 increased Level of IL-1β, IL-6, TNFα and INF-γ decreased IL-4 and IL-10 increased	[81]
45.	*Dictyophora indusiata* (Phallaceae)	Polyphenols and polysaccharides	Balb-C mice	Weight gain (43.5 ± 3 to 38.00 to 3.00 g) decreased significantly (*p* ≤ 0.0001), Epididymal, subcutaneous fat accumulation and liver fat reduced (*p* ≤ 0.01) in dose dependent pattern PPAR-γ, C/EBPα and SREBP-1c and FAS (*p* ≤ 0.001) decreased and ACC increased ALT, AST, TG and FFA decreased (*p* ≤ 0.001) Firmicutes decreased and Bacteroidetes increased	[112]
46.	Whole plant of *Ishige okamurae* (Ishigeaceae)	Diplorethohydroxycarmalol isolated	C57BL/6J male mice	TG (137.88 ± 16.24 to 86.73 ± 11.03 mg/dL), LDL-C (22.24 ± 1.40 to 16.82 ± 2.02 mg/dL), leptin (2.04 ± 0.59 to 1.23 ± 0.37 mg/dL) AST (47.11 ± 6.07 to 41.02 ± 1.52 mU/mL) decreased Expression of AMPK and ACC increased (*p* ≤ 0.01) C/EBPα, SREBP-1c, PPAR-γ and FABP4 (*p* ≤ 0.01) FAS (*p* ≤ 0.05)	[89]
47.	Whole plant of brown algae *Sargassum thunbergii*	Polyphenols of algae	C57BL/6 mice	Body weight gain, epididymal fat, subcutaneous fat, mesenteric fat and perirenal fat decreased (*p* ≤ 0.05) TG (30.65 ± 2.19 to 23.28 ± 1.87 nmol/µL), TC (84.77 ± 4.05 to 73.76 ± 1.61 µg/µL), Leptin (3521.6 ± 0.09 to 2299.6 ± 0.3 pg/mL) insulin (2.21 ± 0.02 to 1.13± 0.42 ng/mL) decreased Expression of UCP1 and UCP3 unregulated. PPAR-γ downregulated	[113]
48.	Red seaweed *Grateloupia elliptica* (Cryptonemiaceae)	Polyphenols, alkaloids, terpenoids, organosulfur compounds, phytosterols, alginates, fucoidans and phlorotannins	Mice and 3T3-L1 adipocytes	Lipids accumulation reduced by 61% at 200 µg/mL Body weight gain suppressed; WAT weight decreased (*p* ≤ 0.05) Expression of SREBP-1 (*p* ≤ 0.001), PPARγ (*p* ≤ 0.0001), FABP-4 (*p* ≤ 0.0001), Expression of UCP-1 and UCP-3 rised TG (110.78 ± 1.62 to 58.22 ± 0.14 mmol/µL), TC (50.17 ± 0.05 to 29.33 ± 0.07 µg/µL), leptin (4308.13 ± 59.37 to 1896.88 ± 1.87 pg/mL), insulin (4.50 ± 0.18 to 3.97 ± 0.00 ng/mL) decreased	[114]
49.	Soybean embryo and enzymatically modified Isoquercetin	Isoflavones, daidzein, glycitein, genisten	C3H10T12 adipocytes and Mice	Body weight gain and fat accumulation decreased significantly Reduced the lipids contents, expression of HSL and PL1N1 increased that increased PKA-dependent cytosolic lipolysis Expression of UCP1, CREB, and SIRT1 increased (*p* ≤ 0.01) at 160 µg/mL	[115]
50.	Fermented fruit extracts of *Diospyros kaki* (Ebenaceae)	Vitamins, polyphenols, dietry fibres, gallic acid, epicatechins, gallocatechins, apicatechins gallate	C57BL/6N mice and 3T3-L1 adipocytes	Body weight gain (15%) and Abdominal fat (27%) and liver mass decreased TC (*p* ≤ 0.01), TG (*p* ≤ 0.05), FBG (*p* ≤ 0.05), LDL-C (*p* ≤ 0.01) decreased and HDL-C (*p* ≤ 0.05) increased Expression of PPAR-γ, C/EBPα, SREBP-1c and FAS decreased AMPK phosphorylation increased	[116]
51	Root extract of *Polygonum cuspidatum* (Polygonaceae)	Resveratrol, Emodin, Picerid-phenolic acids	3T3-L1 adipocytes	Expression of PPAR-γ, C/EBPα, SREBP-1c, aP2, FAS, NF-kβ, p38, p38 mitogen activated protein kinases, JNK significantly decreased at 150 µg/mL and JNK increased (*p* ≤ 0.05)	[92]

**Table 2:** Biologically synthesized metallic nanoparticles and nanoencapsulated secondary metabolites showing anti-obesity efficacy.

Sr. No.	Type of Nanostructure	Chemical Constituents Involved	Characteristics of Nanostructure	Experimental Model	Important Findings	Reference
1.	Nanocellulose from Grape seeds (*Vitis vinifera*)	Pyrogallol, Protocatechuic acid, Chlorogenic, e-vanillic, Benzoic, Naringenin, Hisperidin, Rosmarinic acid, Kaempferol	4–7 nm in width and 37–45 nm in length	Rat	Body weight, weight of liver, kidney, heart and spleen reduced (*p* ≤ 0.05), body weight gain decreased up to 13.73 ± 1.91 and 25.69 ± 2.20% at 2% and 4% nanoparticles diet respectively, Food intake decreased up to 14.00 and 12.00 g/day at 2% and 4% NPs diet respectively, TGs and TC value decreased to 85.97 ± 6.08 and 74.80 ± 3.23 mg/dL at 2% NPs diet	[118]
2.	Gold nanoparticles of *Salacia chinensis*	Saponins, flavonoids and proanthocyanidins	Crystalline, 20 to 50 nm in size and spherical in shape	Rat	Adipose index, leptin and resistin level decreased and adiponectin level increased (*p* ≤ 0.05), BMI and body weight decreased (*p* ≤ 0.05), AMPKα1 and pAMPKα1 decreased	[119]
3.	Gold nanoparticles of *Smilax glabra*	Phenols, alkaloids, flavonoids, glycosides, steroids, tannins, resin and volatile oils	20 nm in size and Spherical in shape	Rat	Body weight and BMI decreased (*p* ≤ 0.001), Leptin and resistin level (*p* ≤ 0.05), adiponectin level increased (*p* ≤ 0.05), plasma insulin level increased (*p* ≤ 0.01), TNFα and IL-1β decreased	[120]
4.	Chitosan NPs and water-soluble chitosan NPs	Chitosan	400–700 nm size of CTS-NPs, 700–1000 nm of WSC-NPs	Rat	Weight gain reduced (*p* ≤ 0.05), epididymal and perirenal WAT decreased, TC, TG, LDL, Lipids accumulation decreased significantly, CBPα and PPAR-γ expression decreased	[121]
5.	Gold nanoparticles of the leaf extract of *Panax ginseng*	Ginsenoside Rh2, Ginsenoside Rg3, Protopanaxatriol saponins and Protopanaxadiol saponins	10–20 nm in size and spherical in shape	3T3-L1 mature adipocytes	Triglycerides accumulation decreased (*p* ≤ 0.001), expression of PPARα, CREBPα and β, SREBP, FAS decreased	[122]
6.	Gold NPs of *Poria cocos*	Triterpenes, glycosides and phenolic acids	Spherical in shape, polydispersed and 20 nm in size	Rat	TC, TG and HDL decreased at the significance level of *p* ≤ 0.05 Leptin, resistin level decreased and adiponectin level increased	[123]
7.	Nanoencapsulated Quercetin	Succinyl chitosan- alginate core shell NPs	91.58 nm in size, spherical shape, 95% EE	Male wistar rat	TG level (51.41%), TC (49.01%), AST (32.69%), ALT (39.36%), ALP (39.15%) decreased respectively	[124]
8.	Nanoencapsulated EGCG	α-tocopherol acetate (8.8%), Kolliphor HS15 (45%), Soy PC (36.2%), EGCG (10%)	104 nm in size and spherical in shape, polydispersed with 95.0% EE	C57BL1/6J mice	TC, HDL-C, TG, Plasma TNF-α, MCP-1, IL-6 decreased significantly at *p* ≤ 0.05, decreased in lesion surface area of aortic arches, secretion of inflammatory factors decreased at 20 µg/mL to 25 mg/kg	[125]
9.	Nanoencapsulated EGCG	1-(Palmitoyl)-2-(5-keto-6-octenedioyl)phosphotidylcholine (Kodia-PC), α-tocopherol	Spherical in shape and 108 nm in size, 96% EE, polydispersity index less than 0.3	Human monocytes THP1 cells	mRNA and protein level of MCP-1 decreased, macrophage EGCG content increased, Kappa β and p38 mitogen-activated protein kinase decreased, PPAR γ decreased, total macrophage cholesterol level decreased	[126]
10	Nanoencapsulated curcumin	Turmeric loaded nanoemulsion (TE-NE), 20 folds lower curcumin content than turmeric extract	136–138 nm in size and spherical in shape, 87.95 ± 0.39 EE	Balb/c mice and HepG2 cells	SREBP-1, PPARγ decreased at 5% of TE-NE, Cleaved caspase-3 and PARP level increased at 300 mg/kg (*p* ≤ 0.01) Body weight gain decreased Total serum cholesterol level decreased (*p* ≤ 0.05), TG level decreased (*p* ≤ 0.01)	[72]
11	Nanencapsulated curcumin	PLA-PEG polymers	Monodispersed, spherical in shape and 117 nm in size with surface charge 35 mV	White albino rats	ALT (63.44%) and AST (54%) decreased NF-kβ, COX-2, TGF-β level decreased and PPAR-α increased	[127]
12	Nanoencapsulated quercetin	Polylactic-co-glycolic acid (PLGA)	179.9 ± 11.2 nm in size, spherical in shape, 0.128 polydispersity index, 86% EE,	Sprague-Dawley diabetic rats	Decreased blood glucose level at 150 mg/kg (*p* ≤ 0.05)	[128]
13	Nanoemcapsulated Resveratrol	Polylactic-co-glycolic acid (PLGA)	Spherical morphology, 176.1 nm in size, 97.25% EE, 14.9% drug loading capacity	HepG2 hepatocytes	Lipids accumulation decreased to 80.77% at 100 µM Decreased hepatocellular differentiation, Decreased TG level in a dose dependent manner (*p* ≤ 0.0001)	[129]
14	Gold nanoparticle (*Dendropanax morbifera* Léveille)	Gold nanoparticle (D-AuNPs)	Spherical (size 10–20 nm)	3T3-L1 adipocytes and HepG2 cells	The adipogenesis process was negatively controlled by D-AuNPs, with downregulated PPARγ, CEBPα, Jak2, STAT3, and ap2 expression in 3T3-L1 cells and FAS and ACC levels in HepG2 cells. So D-AuNPs exert antiadipogenic properties.	Yi, [130]
15	*Trans* Resveratrol-encapsulated NPs (L-R nano)	DSPE-PEG 5000 peptide incorporated L-R nano with ASC targets	ASC-targeted nanoparticle	C57BL/6 mice and 3T3L1 adipocytes	Targeted delivery of browning agents to adipose stromal cells (ASCs) in subcutaneous WAT	[131]

## Abbreviations

**Abbreviation**	**Abbreviation Expanded Form**
ACC	Acetyl Co~A Carboxylase
ALP	Alkaline Phosphatase
ALT	Alanine Aminotransferase
AMPK	Adenosine Monophosphate-Activated Protein Kinase
Ap2	Activating Protein 2
AST	Aspartate Aminotransferase
BAT	Brown Adipose Tissue
BMI	Basal Metabolic Index
C/EBP	CCAAT/Enhancer Binding Proteins
CA	Carnosic Acid
CDIVK	*Cis*-3′,4′-Diisovalerylkhellactone
CNS	Central Nervous System
CPT1A	Carnitine Palmitoyl Transferase 1A
CREB	cAMP Response Element Binding Proteins
CRP	Capsicoside G- Rich Protein
CYP7A1	Cytochrome P_450_ Family 7 Subfamily A Member 1
EGCG	Epigallocatechin’s Gallate
FABP	FAS Binding Proteins
FAS	Fatty Acid Synthase
FBG	Fasting Blood Glucose
FFA	Free Fatty Acids
HDL	High Density Lipoproteins
HFD	High Fat Diet
IL	Interleukin
Jak2	Janus kinase 2
JNK	cJun N-terminal Kinase
LDL	Low Density Lipoproteins
mTOR	Mechanistic Target of Rapamycin
NPY	Neuropeptide Y
ORX	ORX Peptide
PGC1-α	Proliferator Activated Receptor Gamma Coactivator 1-A
PLA-PEG	Polylactic Acid-Polyethylene Glycol
PLGA	Polylactic-Co-Glycolic Acid
PPAR	Peroxisome Proliferator-Activated Receptors
PTP-1B	Protein Tyrosine Phosphtase 1B
SCD-1	Stearoyl-CoA Desaturase-1
SCFAs	Short Chain Fatty Acids
SIRT1	Sirtuin 1
SREBP	Sterol Regulatory Elementary Binding Proteins
STAT 3	Signal Transducer and Activator of Transcription 3
TC	Total Cholesterol
TG	Triglycerides
TNF-α	Tumour Necrosis Factor-Alpha
UCP1	Uncoupling Protein
WAT	White Adipose Tissue
